# Expanding Saccular Aneurysm of the Superficial Femoral Artery Managed With Endovascular Grafting: A Case Report

**DOI:** 10.7759/cureus.78013

**Published:** 2025-01-26

**Authors:** Caleb I Peterson, John Royalty

**Affiliations:** 1 Osteopathic Medicine, Lincoln Memorial University DeBusk College of Osteopathic Medicine, Harrogate, USA; 2 Cardiovascular Surgery, Citrus Memorial Hospital, Inverness, USA

**Keywords:** aneurysm repair, endovascular aneurysm repair, endovascular stenting, endovascular surgical repair, femoral artery aneurysm, peripheral vascular surgery, pseudo aneurysm, saccular aneurysm, superficial femoral artery aneurysm, true aneurysm

## Abstract

Aneurysms in the superficial femoral artery (SFA) are rare, making up a very small percentage of all femoral and peripheral artery aneurysms. Most aneurysms of the SFA are asymptomatic and therefore remain undiagnosed. These are concerning as SFA aneurysms carry higher rates of rupture, which can lead to acute limb ischemia. Previous studies have primarily focused on open vascular repair as a treatment option for SFA aneurysms. However, we present a case where an expanding superficial femoral artery aneurysm was successfully treated with endovascular repair.

## Introduction

Aneurysms in the femoral arteries (FAA) are rare, occurring in roughly five out of 100,000 patients. Of these, only 15-20% are located in the superficial femoral arteries (SFA) [1]. It has been calculated that the distribution of aneurysm incidence in the thigh is 80% in the common femoral artery (CFA), 15% in the superficial femoral artery (SFA), and 5% in the profunda femoris artery (PFA) [1]. Isolated aneurysms of the superficial femoral artery are even rarer, with an incidence rate of 1% for all femoral artery aneurysms and 0.5% for all peripheral aneurysms [2].

The low occurrence is likely due to the anatomical location, deep within the muscular department of the proximal leg [3,4]. Superficial femoral artery aneurysms (SFAA) are often asymptomatic but can present with a variety of clinical signs when symptomatic. These may include a pulsatile mass in the thigh, lower extremity edema, skin changes, and pain in the thigh, groin, or leg [5]. These aneurysms, like all, carry the risk of thrombosis, embolization, or rupture [5]. FAA has been shown to co-occur with other peripheral aneurysms in 27% of cases and abdominal aortic aneurysms (AAA) in 40% of cases [3]. Like other aneurysms, true SFAAs carry similar risk factors including hypertension, hyperlipidemia, smoking, male sex, and diabetes mellitus [6-8]. To note, the cause of true SFAA formation is usually atherosclerotic, typically seen in elderly individuals with a history of smoking and cardiovascular disease. True SFAAs are typically not associated with trauma, as opposed to pseudoaneurysms that are commonly associated with trauma and percutaneous interventions.

## Case presentation

A 65-year-old male presented to the vascular clinic with symptomatic claudication in the distal right lower extremity for a duration of five weeks. The patient described pain in the right calf and ankle with intermittent loss of sensation over the right tibialis anterior. The patient described the pain as sharp and severe, with the only palliative factor consisting of hanging his feet off the bed at night to increase arterial flow. The pain was constant with interference with sleep and activities of daily living (ADL).

**Figure 1:**
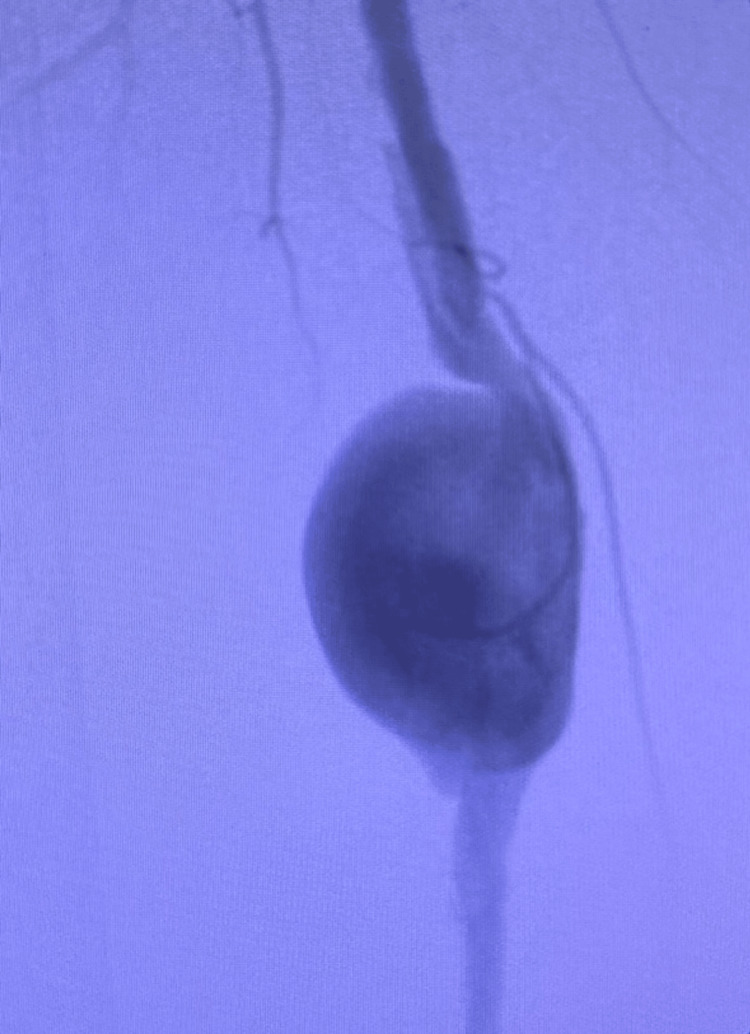
Angiography X-ray with fluoroscopy of right thigh showing 3.2-cm saccular aneurysm dilation of the superficial femoral artery

On initial evaluation, the right medial thigh displayed blue discoloration, with a pulsatile mass roughly 2 cm in diameter felt on superficial palpation. The peripheral arteries were easily palpable except for the right anterior and posterior tibial, which were non-palpable. Prior history was significant for childhood trauma involving a screwdriver inserted into the right medial thigh, and a right knee replacement two years before the initial office visit. The patient also had an outpatient positional atherectomy of the right lower extremity six weeks prior. Five weeks before our initial office visit, a computed tomography (CT) angiogram with runoff showed an isolated 2.2 cm focal saccular aneurysm of the right superficial femoral artery. Another CT angiogram was performed five weeks later that showed focal dilation of the superficial femoral artery aneurysm now measuring 3.2 cm with thrombus deposition (Figure 1, Video 1). On both interpretations, radiologists described focal dilation as saccular aneurysm enlargement. 

**Video 1:** Angiography X-ray with fluoroscopy of right thigh showing 3.2-cm saccular aneurysm dilation of the superficial femoral artery

Due to symptomatic claudication and rapid aneurysm enlargement, an endovascular stent grafting procedure was performed on the right superficial femoral artery and 10,000 units of heparin were administered via intravenous catheter. Ultrasound-guided wire access was obtained in the left common femoral artery with subsequent wire advancement to the distal aorta. A 5 French (Fr) diagnostic catheter was passed into the distal aorta allowing wire access to the right iliac system. After wire access to the right iliac system, the 5 French diagnostic catheter was passed to the right distal external iliac artery and common femoral artery junction. With the 0.035-inch wire placed from the left common femoral artery, a short 5 French sheath was passed over the aortic bifurcation down to the proximal right common femoral artery. A 6 French sheath was then passed from the left common femoral artery over to the proximal right common femoral artery. Through that sheath, a V18 wire was passed from the left common femoral artery to the right popliteal artery. Selective angiograms were then performed from the right groin through the distal lower extremity showing the isolated right superficial femoral artery aneurysm measuring 3.2 cm with thrombus deposition (Video 2). 

**Video 2:** Angiography X-ray with fluoroscopy of right superficial femoral artery showing 3.2-cm saccular aneurysm with wire placement

Over the V18 wire and through the long 6 French sheath, two covered drug-eluting stents were placed, 5 x 10 cm and 6 x 10 cm with a 4 cm overlap. The final angiogram showed no evidence of the aneurysm or extravasation from the stent sites (Video 3). Postoperatively, a posterior tibial artery pulse was present on the right lower extremity. 

**Video 3:** Angiography X-ray with fluoroscopy of right superficial femoral artery with focal stent placement

To date, the patient has had a successful postoperative course. At three weeks post-repair the patient was without ischemic or embolic complications. Ultrasound duplexes performed at three weeks follow-up revealed biphasic signals in the posterior tibial artery and dorsalis pedis artery on the right lower extremity. Five months post-repair the patient remained asymptomatic with no claudication present in the right lower extremity. A right lower extremity arterial Doppler at three months post-repair showed biphasic signals down to the tibial peroneal artery. The posterior tibial artery was monophasic proximally and biphasic distally. His anterior tibial artery was biphasic throughout. The dorsalis pedis artery was monophasic. Stenting in the superficial femoral artery remained patent across the aneurysm with no leakage in the aneurysm sac. Biphasic signals were seen throughout the popliteal and superficial femoral arteries.

## Discussion

Aneurysms of the SFA are rare and often go undiagnosed due to their anatomical location deep within the muscular canal of the leg [3,4]. They are usually discovered incidentally, except for cases of acute ischemia, embolization, thrombosis, or rupture, which requires immediate surgical evaluation. The leading symptom associated with SFAA is rupture with a rate of 30-50%, which is much higher than other peripheral aneurysms [1,9]. Surgical repair is indicated in all symptomatic patients, whether for reducing ischemic pain, preventing distal emboli, preventing rupture, or increasing arterial flow [9]. There is no current consensus for asymptomatic aneurysms, however, most authors will recommend repairing aneurysms larger than 2.5 cm [9]. 

Several surgical approaches are described in the literature, with each method providing its own advantages and disadvantages. Surgical repair methods for FAA are broadly divided into two categories, open repair, and endovascular repair (Table 1). Open repair typically involves the exclusion of a bypass graft or placement of an interposition graft and should be performed in the setting of infection, arteriovenous fistula presence, or ischemic soft tissue changes [1,6]. In a study performed by Perini et al., open surgical repair for isolated superficial femoral artery aneurysms reported limb salvage and graft patency rates of 88%, and 85%, respectively. Endovascular repair is suitable for both true and false aneurysms of the SFA. The advantages of endovascular repair include shorter recovery periods, aneurysm exclusion, and the creation of an endoluminal bypass site [1,5,9-11]. To date, endovascular repair has been rarely researched in SFAA aneurysm repair [12-14].

**Table 1:** Comparison of surgical treatment options for femoral artery aneurysms

Treatment Options	Advantages	Disadvantages	First-Line Treatment
Open repair	Long-established and well-documented treatment.	Invasive with longer hospital stays.	Large or complex femoral aneurysms requiring bypass or ligation.
	Effective for complex aneurysms: Particularly suitable for large or multiple arterial branches.	Higher risk of surgical complications such as infection, bleeding, or thromboembolic events.	Aneurysms with extensive thrombus or involvement of critical side branches.
	Can address aneurysms involving critical side branches of arteries.	Requires general anesthesia.	Usually, first line in setting of infection, arteriovenous fistulas or soft tissue changes.
Endovascular repair	Performed through small incisions or punctures, resulting in a shorter hospital stay (1-2 days).	Limited effectiveness for very large, complex, or heavily calcified aneurysms, or those with challenging anatomy.	Popliteal aneurysms in patients with favorable anatomy or high surgical risk.
	Lower perioperative morbidity: Reduced trauma, pain, and fewer immediate complications.	Potential for endoleaks or graft-related complications: Risk of blood continuing to flow into the aneurysm sac (endoleak) or graft migration/failure.	Feasible for both true and false aneurysms of the SFA.
	Faster recovery and reduced pain.	Depends on having appropriate arterial anatomy and a landing zone for the stent graft.	
Hybrid approach	Combines open and endovascular techniques, offering a tailored treatment for complex aneurysms	Requires highly skilled surgical teams and specialized facilities.	Complex femoral or popliteal aneurysms unsuitable for purely open or endovascular repair.
	Can treat aneurysms unsuitable for either open or endovascular repair alone.	Potentially longer operative times and recovery periods compared to pure endovascular repair.	Cases where branch preservation is critical.
	Minimizes invasiveness in some cases while addressing anatomical challenges.	Risks and complications of both open and endovascular techniques may be present.	

## Conclusions

Isolated superficial femoral artery aneurysms are rare, but carry the complications of thrombosis, embolization, or rupture. It is important to carry a high index of suspicion as rupture is associated with increased morbidity and mortality. Risk factors for true SFA aneurysms include hypertension, smoking, male sex, and diabetes. When diagnosed, symptomatic cases or asymptomatic individuals with rapid enlargement should be evaluated for surgical candidacy. Research involving stent grafts in the SFA is limited yet has shown promising results in emerging literature. The use of endovascular grafting for SFA aneurysms has been shown to provide positive treatment outcomes and should be studied as an alternative to open repair.
